# Does cervical curvature affect neurological outcome after incomplete spinal cord injury without radiographic abnormality (SCIWORA): 1-year follow-up

**DOI:** 10.1186/s13018-022-03254-7

**Published:** 2022-07-26

**Authors:** Can Qi, Junming Cao, Hehuan Xia, Dechao Miao, Yaming Liu, Junfei Guo, Zequn Li, Zhiyong Hou

**Affiliations:** 1 Department of Orthopaedic Surgery, Third Hospital of Hebei Medical University, Shijiazhuang, Hebei Province China; 2 Key Laboratory of Biomechanics of Hebei Province, Third Hospital of Hebei Medical University, Shijiazhuang, Hebei Province China; 3 Department of Spinal Surgery, Third Hospital of Hebei Medical University, Shijiazhuang, Hebei Province China; 4 The Department of Radiology, Third Hospital of Hebei Medical University, Shijiazhuang, Hebei Province China; 5 NHC Key Laboratory of Intelligent Orthopaedic Equipment, Shijiazhuang, Hebei Province China

**Keywords:** Cervical spine curvature, SCIWORA, Nonsurgical treatment, Spinal cord injury, Correlation analysis, Clinical value

## Abstract

**Background:**

At present, surgery is the primary clinical treatment for SCIWORA patients, but conservative treatment still plays an important role in patients with incomplete spinal cord injury. As an important index of cervical spine degeneration, cervical curvature has an impact on the prognosis of spinal cord injury patients. This paper studied the prognosis of conservatively treated patients with SCIWORA and the correlation between cervical curvature and neurological prognosis.

**Methods:**

A retrospective study was conducted in all the patients with SCI admitted to the Third Affiliated Hospital of Hebei Medical University between January 2017 and June 2020. Data were recorded in 106 eligible patients, including sex, age, injury factors, Cobb angle, CCI, CSA, and ASIA motor and sensory scores. The Wilcoxon sign rank sum test was used to analyze the data postinjury and at the 1-year follow-up. Pearson correlation analysis was performed for the Cobb angle, CCI and CSA. Simple linear regression analysis and multiple linear regression analysis were performed for each group of variables.

**Results:**

The Wilcoxon signed rank sum test confirmed that the Cobb angle, the CCI and the CSA of the patients were not significantly different at the 1-year follow-up when compared with the postinjury values, and the ASIA motor and sensory scores were significantly improved. The Pearson correlation analysis showed correlations among the Cobb angle, the CCI and the CSA. Simple linear regression analysis and multiple linear regression analysis showed that the nerve recovery rate was negatively correlated with age and was positively correlated with the Cobb angle.

**Conclusion:**

Conservative treatment of incomplete SCIWORA can achieve a good prognosis. There is a clear correlation between the Cobb angle, CCI and CSA, and the Cobb angle, as an important influencing factor, needs to be considered. For SCIWORA patients undergoing nonsurgical treatment, improving cervical curvature is beneficial to the prognosis of patients. Age negatively affects the neurological prognosis.

## Introduction

Pang et al. [1] first proposed the concept of spinal cord injury without radiographic abnormality (SCIWORA) in 1989. In recent years, with the continuous improvement of MRI technology, the clinical evaluation and understanding of SCIWORA have significantly evolved [2]. In a retrospective analysis, Tewari et al. [3] found that patients with SCIWORA accounted for up to 12% of patients with traumatic spinal cord injury, and most of these patients had incomplete spinal cord injury. Therefore, the treatment of spinal cord injury without radiographic abnormalities has always been controversial. At present, there is still a lack of consensus on the ideal treatment of SCIWORA. The detailed and accurate imaging classification evaluation has clinical value in guiding the choice of treatment for these patients [4, 5]. In recent years, several studies have been conducted on the injury mechanism and clinical treatment of SCIWORA. New findings, new diagnoses and treatment techniques are evolving, management methods are ever changing, and the debate between surgical treatment and conservative treatment is ongoing [6].

Normal cervical curvature plays an important role in maintaining effective motor function, and a series of vertebral biomechanical imbalances caused by abnormal cervical curvature may lead to further development of cervical degenerative diseases [7]. Cervical curvature is not obvious in infancy but becomes more or less fixed in adulthood as bone develops. Lateral cervical radiographs were initially used as a basic method for measuring cervical curvature and as a reference index for determining treatment plans and evaluating efficacy. At present, cervical curvature is mainly used in evaluating patients with cervical spondylosis, but there are few studies on the relationship between cervical curvature and spinal cord injury [8, 9]. Studies on the prognosis of spinal cord injury mainly focus on MRI findings, injury segment, ASIA grade, spinal canal to vertebral body ratio, trauma mechanism, degenerative changes around the intervertebral disk and other factors, but there are few studies on the influence of cervical curvature changes [10, 11]. Patwardhan et al. [12] studied the effect of cervical curvature on surgical outcome and nerve recovery, but there are few reports on the nonsurgical treatment of spinal cord injury.

This research aims to study the conservative treatment outcomes in patients with incomplete SCIWORA and the correlation between cervical curvature and prognosis in SCIWORA patients. In this study, the correlation between nerve improvement and cervical curvature was analyzed to further verify whether cervical curvature is a suitable index for spinal cord injury.

## Data and methods

**Fig. 1:**
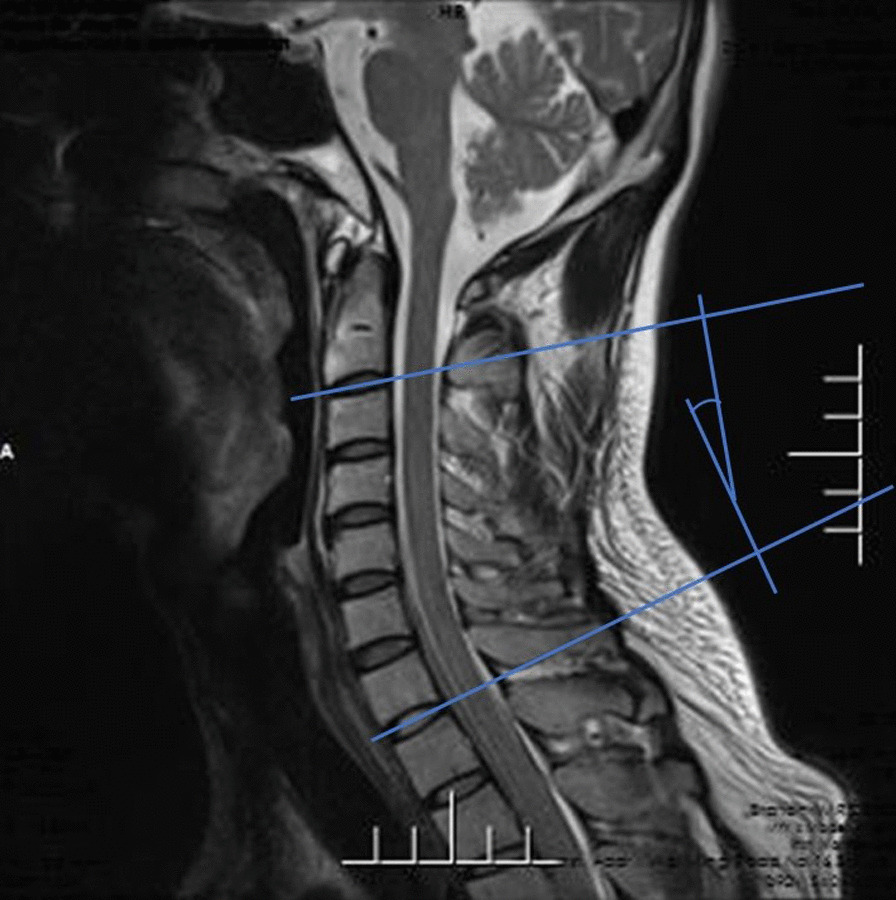
For the extension lines of the lower endplates of C2 and C7, draw the perpendicular lines of the two lines, and the acute angle formed by the intersection of the two lines is the Cobb angle

**Fig. 2:**
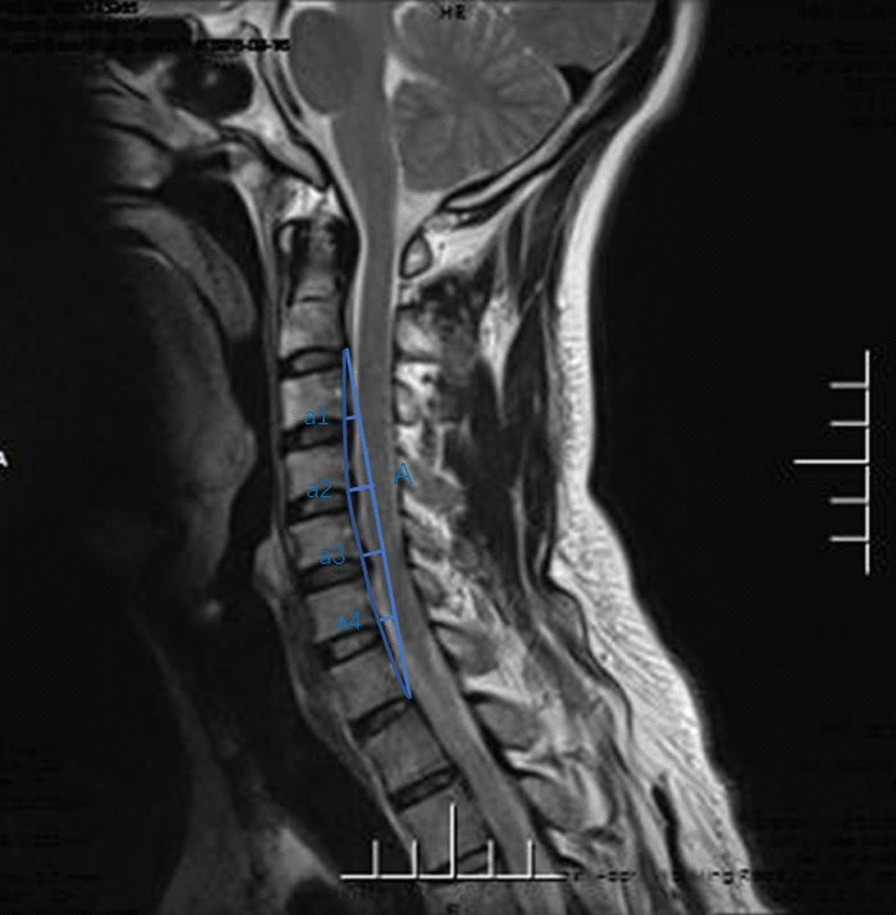
The line connecting the posterior and lower edges of the C2 and C7 cervical vertebrae is line A, and the vertical lines from the posterior lower edge of the C3 to C6 vertebra to Line A are a1, a2, a3 and a4. CCI = (a1 + a2 + a3 + a4)/A * 100%

**Fig. 3:**
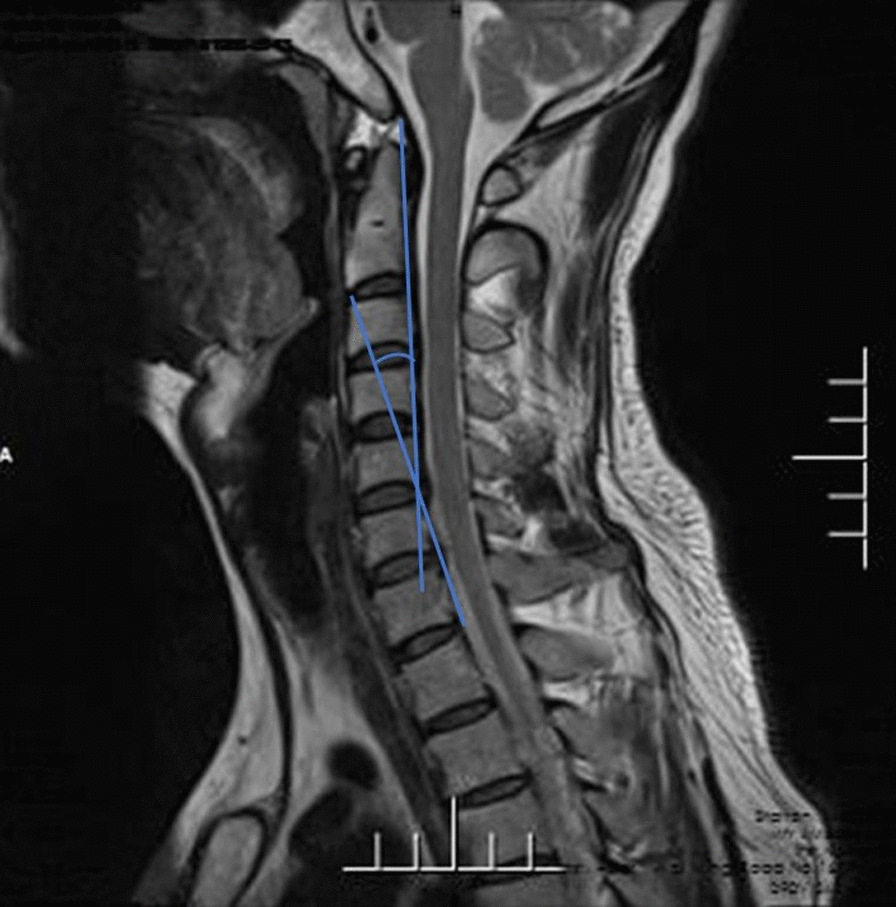
Parallel lines of the posterior margins of the C2 and C7 vertebral bodies. The acute angle at which it intersects is the cervical spine angle (CSA)

A retrospective study was conducted on the data of all the adult patients with SCI admitted to the Third Affiliated Hospital of Hebei Medical University from January 2017 to June 2020. The 623 hospitalized patients were identified as having spinal cord injuries according to the International Classification of Diseases, Ninth Revision. The International Classification of Diseases is designed to promote international comparability in the collection, processing, classification, and presentation of mortality statistics. The following exclusion criteria were used: (1) age ≤ 18 years old; (2) radiographic evidence of trauma (fracture, dislocation or subluxation); (3) SCI of the thoracic or lumbar spine; (4) history of spinal pathological changes or spinal surgery; (5) complications including brain, chest, abdominal injury or limb trauma; (6) surgical treatment was ultimately administered; (7) patients with mental disorders or other factors who were unable to cooperate. After the eligible patients were admitted to the hospital, they received a fixed neck collar. Other interventions included strategies to reduce swelling and steroid treatment. All patients needed a fixed neck collar or cervical collar for at least 3 months and were instructed to limit high-risk activities for 6 months. Patients were examined every 2 months to determine whether the patient had cervical instability. Symptoms were exacerbated during the observation period, and MRI showed that surgical intervention was needed [13]. A total of 106 patients, between the ages of 23 years and 81 years, met the study requirements, with an average age of 50.5 years. In this study, the data were measured by two attending physicians with more than 10 years of experience in the spinal field. After each doctor performed the measurements, the average value of the two measurements were taken to reduce errors. If there was a large difference in the data, then the third surveyor completed the evaluation. All measurements were obtained on MRI. The measurement data included the Cobb angle, the cervical curvature index (CCI), and the cervical spine angle (CSA). Cobb angle: For the extension lines of the lower endplates of C2 and C7, draw the perpendicular lines of the two lines, and the acute angle formed by the intersection of the two lines is the Cobb angle (Fig. 1). CCI: The line connecting the posterior and lower edges of the C2 and C7 cervical vertebrae is line A, and the vertical lines from the posterior lower edge of the C3 to C6 vertebra to Line A are a1, a2, a3 and a4. If the posterior lower edge of C3-C6 is located on the dorsal side of line A, the value of A is recorded as negative. CCI = (a1 + a2 + a3 + a4)/A * 100% (Fig. 2). CSA (Harrison's method): Parallel lines of the posterior margins of the C2 and C7 vertebral bodies. The acute angle at which it intersects is the cervical spine angle (Fig. 3). Length data are easily affected by factors such as the height of the patient, enlargement or reduction on imaging examination, etc. Angle and proportion data are more objective and accurate [14].

The American Spinal Injury Association (ASIA) score was obtained to determine the neurological function of the patients. Motor score: Motor function score of 10 pairs of key muscles in the upper and lower limbs, 5 points for each muscle, with a total score of 100 points; Sensory score: The left and right 56 sensory scores, including light touch and needling sensation, 4 points for each, with a total score of 224 points. Neurological recovery rate = (ASIA motor and sensory scores at the follow-up—ASIA motor and sensory scores postinjury)/(324-ASIA motor and sensory scores postinjury) 100%.

The SPSS version 21.0 statistical software (IBM, Armonk, NY, USA) was used for statistical analysis. The Kolmogorov–Smirnov test was used to verify whether the data conformed to the normal distribution.

## Results

### Patient population and disposition

The 106 patients who met the inclusion criteria were characterized as 68 cases of fall injury (64.15%), 26 cases of car accident injury (24.53%), 10 cases of heavy object smashing injury (9.43%), and 2 cases of traction injury (1.89%). Fall injury was the main injury factor for SCIWORA patients. In this study, all 106 patients with incomplete SCI had ASIA Impairment Scale [9] grade C or D at admission, and 18 of them had AIS grade E at the 1-year follow-up. All patients underwent cervical X-ray, CT and MRI examinations after admission. MRI scanning results of 106 patients showed no obvious spinal cord compression, no spinal canal stenosis, and no MRI abnormalities in or outside the spinal cord. Seven cases of cervical kyphosis. During the study period, 623 hospitalized patients were identified as having spinal cord injuries. Of these, 22 and 43 patients were excluded because they had spinal cord injuries at C1-2 and the thoracic or lumbar region, respectively. Thirty-one patients had complete spinal cord injuries and were categorized as AIS Grade A. In addition, 59 patients were excluded from the analysis because they had a craniocerebral or thoracoabdominal injury. Of the remaining 468 patients, only 424 underwent cervical X-ray, CT, or MRI examination. A total of 197 patients diagnosed with fractures by CT scan were excluded, so the data analysis included the remaining 227 patients diagnosed with SCIWORA. Of these, 119 patients eventually underwent surgery, and 2 patients were lost to follow-up. The follow-up period was 1 year. Notably, none of the patients treated in this study developed serious complications (Table 1).

**Table 1:** Comparative analysis of postinjury and 1-year follow-up data

	Post-injury	1-Year follow-up	*P*
Cobb angle	15.07 ± 10.67	15.39 ± 10.10	0.34
CCI	0.15 ± 0.11	0.15 ± 0.11	0.14
CSA	19.93 ± 10.89	19.94 ± 10.87	0.40
ASIA motor and sensory score	255.08 ± 19.95	307.43 ± 15.03	< 0.05

*Values are expressed as the mean ± SD. *P* < 0.05 was considered statistically significant

### Nonparametric test

The Kolmogorov–Smirnov test showed that age, Cobb angle, CCI and CSA data did not conform to a normal distribution. The Wilcoxon sign rank sum test was used to analyze Cobb angle, CCI and CSA postinjury and at the 1-year follow-up, and the results were not statistically significant. The postinjury and 1-year follow-up ASIA motor and sensory scores were analyzed (*P* < 0.05). The neurological function of SCIWORA patients was significantly improved after conservative treatment, and 18 patients achieved complete recovery. Conservative treatment could benefit patients with SCIWORA (Table 2).

**Table 2:** Simple linear regression analysis results

	*F*	*P*	Durbin–Watson	Adjusted *R*^2^
Age	2.14	0.15	1.84	0.011
Sex	0.84	0.36	1.81	− 0.002
Cobb angle	40.79	< 0.05	1.68	0.275
CCI	38.03	< 0.05	1.65	0.261
CSA	33.13	< 0.05	1.76	0.234
ASIA motor and sensory score postinjury	0.109	0.74	1.80	− 0.009

*P < 0.2 was included in multiple linear regression analysis

### Pearson correlation analysis

Pearson correlation analysis was conducted for the Cobb angle, CCI and CSA, in which the Cobb angle and CCI analysis result was 0.91, the Cobb angle and CSA analysis result was 0.90, the CCI and CSA analysis result was 0.88. There was a positive correlation among the three groups of cervical curvature, Cobb angle, CCI and CSA.

### Simple linear regression analysis

**Fig. 4:**
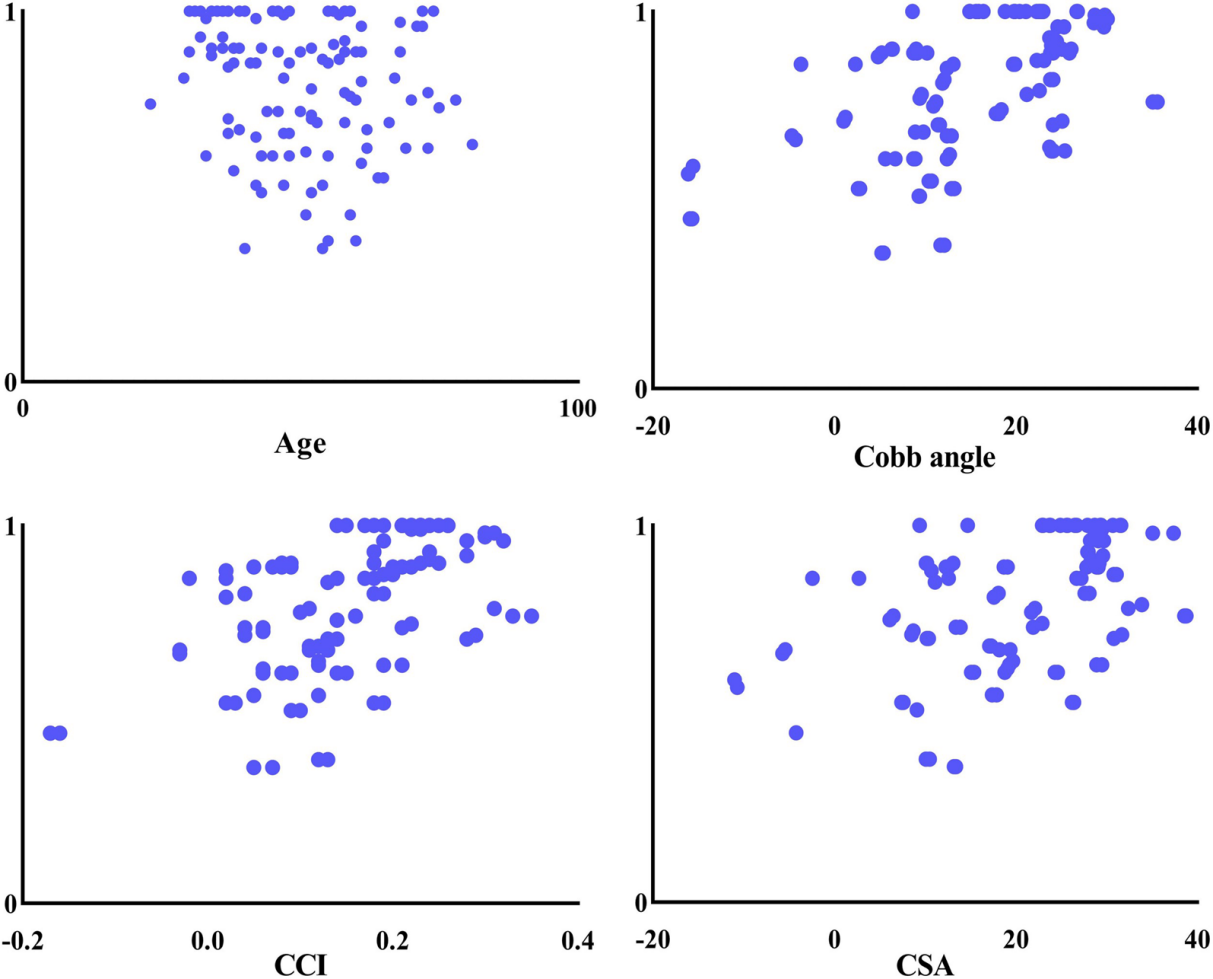
Simple linear regression results showed that there was a positive correlation between the neurological recovery rate and the Cobb angle, the CCI and the CSA but no significant correlation with age

Sex, age, Cobb angle, CCI, CSA, ASIA motor and sensory scores, postinjury and neurological recovery rates were analyzed by simple linear regression. The observed values in this study are independent of each other. The results showed that there was no significant correlation between the neurological recovery rate and sex or age. There was a linear correlation between the nerve function recovery rate and the Cobb angle, CCI and CSA, and the Cobb angle had a more significant correlation (*F* = 40.79, adjusted *R*^2^ = 0.275) (Fig. 4). The Cobb angle could explain 27.5% of the variation in the nerve recovery rate, further indicating that among the indices of cervical curvature, the Cobb angle was more closely related to the neurological recovery rate (Table 2).

### Multiple linear regression analysis

**Fig. 5:**
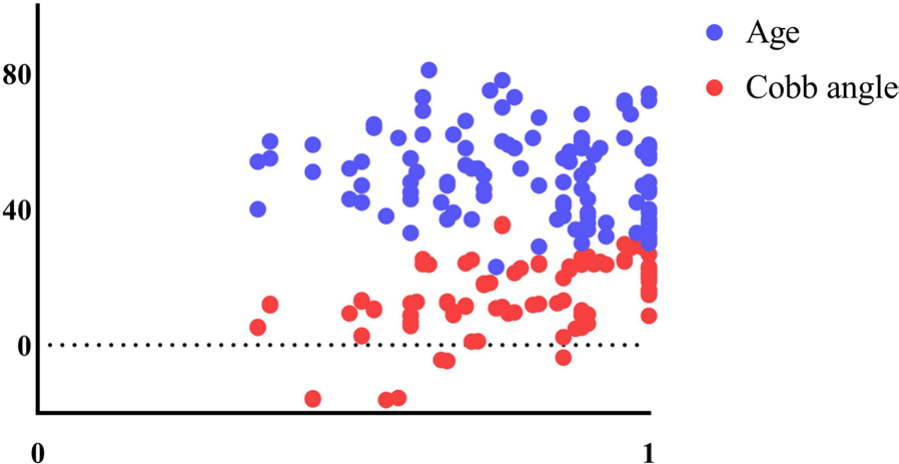
Multiple linear regression results showed that the rate of neurological improvement was positively correlated with the Cobb angle and negatively correlated with age, but the correlation was not significant

According to the simple linear regression analysis, *P* < 0.2 was used as the inclusion criterion, and age, Cobb angle, CCI, and CSA were included in the multiple linear regression analysis (adjusted *R*^2^ = 0.313, *F* = 12.96, *P* < 0.05). The final results showed that there was a certain correlation among the neurological recovery rate, age and the Cobb angle. There was a negative correlation with age and a positive correlation with the Cobb angle. These variables accounted for 31.30% of the variation in the neurological recovery rate (Table 3). In the simple linear regression analysis, there was no significant correlation between neurological recovery rate and age. However, CCI and CSA were positively correlated with the neurological recovery rate in the simple linear regression, which did not indicate an obvious correlation in the multivariate linear regression analysis. After removing the Cobb angle, the results showed that the neurological recovery rate was positively correlated with age and the CCI. After removing the Cobb angle and the CCI, the results showed that there was a certain correlation among the neurological recovery rate, age and the CSA. The Cobb angle, CCI and CSA are correlated with each other. Therefore, confounding variables were generated after the data of the three groups were recorded in the multiple linear regression analysis. As a confounding variable, age should be included in the multiple linear regression analysis. The results show that the older the age is, the worse the neurological recovery rate, but the correlation is not high. The Cobb angle, CCI and CSA groups of independent variables affected the results of the multiple linear regression analysis due to the linear relationship, but the comprehensive evaluation showed that the Cobb angle was the main influencing factor, and correcting the Cobb angle in conservatively treating patients with SCIWORA could help improve the neurological recovery rate (Fig. 5).

**Table 3:** Multiple linear regression analysis results

	*B*	*β*	*t*	*P*	*F*	Adjusted *R*^2^
Age	− 0.003	− 0.23	− 2.773	0.01	12.958	0.313
Cobb	0.008	0.49	2.072	0.04		
CCI	0.260	0.17	0.817	0.42		
CSA	− 0.001	− 0.08	− 0.384	0.70		

*P < 0.05 was statistically significant

## Discussion

The mechanism of SCIWORA has been well studied, and most of cases are caused by cervical hyperextension injury [15]. There are many studies on the treatment of SCIWORA, and surgery is still the preferred treatment for SCIWORA patients. Kawano et al. [16] found that surgical treatment was not beneficial when compared with conservative treatment, and conservative treatment was also suitable for SCIWORA patients. The 106 patients in this study experienced an improvement in neurological function with conservative treatment, which is consistent with Kawano's study and confirms that SCIWORA patients benefit from conservative treatment. Maeda et al. [17] confirmed that neurological improvement in SCIWORA patients was mainly related to the initial injury mechanism, the spinal canal diameter, patient age, the injury degree, the presence of disk-ligament injury and the severity of the neurological syndrome, therefore, guiding further research on the surgical indications of SCIWORA and its related prognosis. However, the surgical indications for SCIWORA may require a deeper consideration. Surgical treatment should be reserved for patients with clear MRI evidence of extraneural findings, including spinal cord compression, ligamentous injury, and instability, along with worsening neurological symptoms or lack of improvement [18]. All 106 patients in this study had incomplete SCIs, and surgical treatment is still recommended for patients with complete SCIs.

The focus of cervical curvature research has ranged from the original correction of developmental malformations to cervical degenerative degeneration, and the current research focus is to determine whether surgical treatment is needed to restore a normal cervical curvature. Many methods have been developed to measure cervical curvature, but a unified standard on the ideal cervical curvature measurement is unavailable. In clinical practice, there are many methods for cervical curvature measurement [19]. In this study, cervical curvature values were measured on MRI, without reference to traditional X-ray lateral measurement. The main reason is that patients with a cervical spinal cord injury need to be immobilized by neck brace, and early standing examination is not possible. In addition, a standing X-ray sometimes fails to fully expose the C6-7 vertebral body, which affects the measurement results. Jun et al. [20] conducted a study on the correlation between the MRI and X-ray measurements of cervical curvature, and the results confirmed that the MRI measurements were also feasible. Regarding the influence of position on cervical curvature, Wang et al. proposed that the numerical measurement of cervical curvature on MRI performed in the supine position was more consistent with the condition of the patients' cervical spine during surgery, thus providing a reference for the improvement of the patients' cervical curvature during surgery [21]. The authors considered that in the early stage of a spinal cord injury, patients are mainly bedridden, so the measurement obtained with the patient in the supine position is closer to the actual cervical vertebral status.

In this study, there were correlations among the three groups of cervical curvature values, and the repeatability, accuracy and reliability of each value were compared, which were similar to the results of other studies [22–26]. However, among the 106 patients, there were no "S"-shaped cervical vertebrae or those with definite scoliosis and kyphosis, and the correlation between cervical curvature values in these patients needs further study. Cervical curvature is mainly used for cervical lateral curvature and cervical degeneration. Suda et al. [27] suggested that the cervical spine sequence with kyphosis was considered abnormal and adversely affected the recovery of cervical nerve function. Further studies by Mohanty et al. combined the sagittal parameters of the cervical spine and found that changes in cervical curvature and sagittal parameters were correlated with neurological prognosis [28]. However, Glassman et al. [29] conducted a study on postoperative sagittal imbalance of the cervical spine and believed that postoperative cervical curvature changes were related to postoperative pain, neurological recovery and the neck disability index (NDI). Liu et al. [30] found that surgery to restore cervical curvature to the normal range was beneficial to the spinal cord drifting backward and effectively reduced the occurrence of axial symptoms. Several studies have suggested that the recovery of cervical curvature is one of the surgical treatment objectives. The recovery of cervical curvature improves the surgical effect and obviously influences nerve prognosis and neck pain relief [31–34]. The authors disagree that surgical treatment improves cervical curvature, relieves spinal cord compression or enhances spinal stability, which may be the root cause of neurological improvement rather than cervical curvature improvement. Further research is needed on whether surgical improvement of cervical curvature will affect surgical results. It is important to note that cervical curvature in patients with total spinal balance is also affected by nonsurgical treatment, so whether the cervical vertebra is simple convex or straight because of cervical spondylosis remains controversial. The cervical curvature is closely related to the overall sagittal balance of the spine. Sagittal imbalance of thoracic or lumbar vertebrae will affect the parameters of the cervical sagittal position, which has guiding significance for spinal orthopedics. [35–38]. In this study, the correlation between the Cobb angle, the CCI, the CSA and the nerve improvement rate was studied. The results showed that the Cobb angle, the CCI, and the CSA all had a positive effect on nerve prognosis, but after a comprehensive analysis, the Cobb angle had a greater effect, which may be related to the correlation between the Cobb angle, the CCI, and the CSA. Age influences the improvement of neurological function, so for patients treated with surgery, the older the patient, the better the neurological function, which may be related to the injury mechanism of the patient. The younger the patient, the greater the injury energy, and neurological recovery is poor [39]. None of the 106 patients in this study had a high energy injury, and the data showed that older patients had poor neurological improvement. However, the influence of age on patients undergoing surgery needs further study.

## Conclusion

For some patients with SCIWORA, conservative treatment can achieve a good therapeutic effect. The prognosis of spinal cord injury worsens with age. There was a clear correlation between the Cobb angle, the CCI and the CSA. The Cobb angle, as an independent risk factor, should be considered and has a positive effect on the prognosis of spinal cord injury within a certain range. For patients with SCIWORA, improvement of the cervical curvature is beneficial to the patients’ prognoses. The clinically abnormal manifestations of cervical curvature are mainly straightness or kyphosis of the cervical curvature, and there are few patients with excessive lordosis of the cervical spine. Further research is needed to determine whether excessive lordosis will affect the neurological prognosis.

## Data Availability

The datasets used and/or analyzed during the present study are available from the corresponding author on reasonable request.

## Abbreviations

SCIWORA: Spinal cord injury without radiographic abnormality
CT: Computed tomography
MRI: Magnetic resonance imaging
ASIA: American Spinal Injury Association
AIS: ASIA Impairment Scale
CCI: Cervical curvature index
CSA: Cervical spine Angle
NDI: Neck Disability Index
